# In the Eyes of the Future: Eye Movement during Near and Distant Future Thinking

**DOI:** 10.3390/vision8020032

**Published:** 2024-05-10

**Authors:** Mohamad El Haj, Ahmed A. Moustafa

**Affiliations:** 1 CHU Nantes, Clinical Gerontology Department, Bd. Jacques Monod, 44093 Nantes, France; 2 LPPL—Laboratoire de Psychologie des Pays de la Loire, Faculté de Psychologie, Université de Nantes, Chemin de la Censive du Tertre, BP 81227, CEDEX 3, 44312 Nantes, France; 3 School of Psychology, Faculty of Society and Design, Bond University, Gold Coast, QLD 4226, Australia; 4 Department of Human Anatomy and Physiology, The Faculty of Health Sciences, University of Johannesburg, Johannesburg 2006, South Africa

**Keywords:** eye movement, future thinking, imagery, mental imagery, temporality

## Abstract

Research has suggested that near future events are typically viewed from a first-person (an own-eyes, also known as field) perspective while distant future events are typically viewed from a third-person (an observer) perspective. We investigated whether these distinct mental perspectives would be accompanied by distinct eye movement activities. We invited participants to imagine near and distant future events while their eye movements (i.e., scan path) were recorded by eye-tracking glasses. Analysis demonstrated fewer but longer fixations for near future thinking than for distant future thinking. Analysis also demonstrated more “field” mental visual perspective responses for near than for distant future thinking. The long fixations during near future thinking may mirror a mental visual exploration involving processing of a more complex visual representation compared with distant future thinking. By demonstrating how near future thinking triggers both “field” responses and long fixations, our study demonstrates how the temporality of future thinking triggers both distinct mental imagery and eye movement patterns.

## 1. Introduction

In daily life, individuals dedicate significant time to envisioning the future. This future-oriented cognition aids in decision-making by offering a mental platform for evaluating, adjusting, and optimizing decisions, thereby enhancing problem-solving abilities and action planning [1,2,3,4]. Alternatively, future thinking may simply provide the ability to cope with the difficulties of daily life [5]. Beyond its practical functions, future thinking also serves an identity function, fostering a sense of self-continuity [6,7,8].

Regarding its temporality, future thinking allows simulating near and distant events. As we emphasize below, research has demonstrated that a key difference between near and distant future thinking is the mental visual perspective. More specifically, near distant future thinking typically implies an “own eyes visual perspective” while distant future thinking typically implies an “outside visual perspective”. Building on this research, we investigate, for the first time, differences in eye movement patterns as associated with the construction of near and distant future events. By doing so, we hope to understand whether mental visual perspectives during different future thinking timescales may be accompanied by specific eye movement patterns.

Several studies have investigated mental visual perspectives for near vs. distant future thinking. These studies were inspired by the assumption that, when imagining ourselves across time, we can use a first-person (i.e., field) perspective involving looking through one’s own eyes, or a third-person (i.e., observer) perspective involving looking at oneself from the outside [9]. The relationship between these two visual perspectives and future thinking was evaluated by D’Argembeau and Van der Linden [10], who invited participants to imagine an event that might happen in the near future (i.e., in the next year,) and another event that might happen in the distant future (i.e., in five to ten years). In each event, participants were invited to report whether they mentally “saw” the scene from their own perspective (i.e., field perspective) or “saw” themselves in their representation (i.e., observer perspective). Results demonstrated that near future events triggered more field perspective responses compared to distant events. Similar results were reported by Macrae et al. [11], who investigated whether perceivers use a different mental perspective when imagining their selves in the near and distant future. Results demonstrated that, whereas near future self-images were dominated by a first-person perspective, distant self-images were dominated by a third-person perspective. In a similar vein, Hamilton and Cole [12] invited participants to imagine an ideal-self that they would like to be in the near or distant future. Results demonstrated that the third-person perspective increased with future temporal distance. Similar findings were reported by Aikman et al. [13], who invited participants to imagine near or distant future events. The authors found that near future events were mainly associated with a first-person perspective. Together, research has consistently reported that, compared to their distant counterparts, near future events are typically viewed from a first-person perspective. Building on this research, we investigated whether different eye movement activities can be observed between distant and near future thinking.

In addition to drawing from previous research on mental visual perspective during future thinking, our study was inspired by investigations into eye movements during future thinking. El Haj and Lenoble [14] conducted a study where participants were asked to imagine future events and recall past events, while their eye movements (scan path) were recorded using an eye-tracker. Results revealed that future thinking elicited fewer fixations and saccade counts compared to past thinking. The authors attributed these fixations and saccades, observed during both past and future thinking, to mental imagery and the visual system’s attempt to locate (via saccades) and activate (via fixations) stored mental representations. While the study of El Haj and Lenoble [14] was the first to investigate eye movement during future thinking, it did not examine the temporal distance (near vs. distant) or visual perspective (first- vs. third-person perspective) of these events.

The link between eye movement and mental imagery has been explored in studies investigating eye movement during past reminiscence. In a groundbreaking investigation, El Haj et al. [15] tasked participants with recalling past events and, in a control scenario, with performing a counting task aloud, while tracking their eye movement patterns with an eye-tracker. Their findings revealed a reduction in the number of fixations but an increase in the quantity, length, and duration of saccades during past reminiscence compared to the counting task. El Haj, Delerue, Omigie, Antoine, Nandrino, and Boucart [15] attributed eye movement activity during past thinking to visual imagery as required to generate the visual scene of the retrieved events. A similar assertion was posited by another study, linking the duration of fixations and saccades during the retrieval of emotional memories to the vividness of these recollections [16]. This connection between eye movement and mental imagery, as illuminated by research on eye movements during past and future reminiscence, can be grounded in the notion that autobiographical memories manifest in the form of visual mental images [17]. This proposition finds support in studies demonstrating superior autobiographical recall in individuals with strong visual imagery compared to those with weaker imagery [18], as well as neuropsychological investigations revealing autobiographical memory impairments in patients with lesions in vision-related brain regions, such as the right occipital lobe at the temporo-occipital junction [19].

To summarize, while previous research has investigated eye movement during future thinking [14], this prior research did not investigate the temporal distance (i.e., near vs. distant) nor the visual perspective (i.e., first- vs. third-person perspective) of these events. This comparison is worth consideration because research has consistently demonstrated that construction of near and distant events involves distinct mental visual perspectives [10,11,12]. Building on this prior research, we investigated whether the first-person perspective, as may be expected for near future thinking, may be accompanied by distinct eye movement pattern (e.g., long fixations). If this is the case, our study will demonstrate for the first time how the visual perspective of future thinking can be accompanied by distinct eye movement activities.

## 2. Method

### 2.1. Participants

The sample consisted of forty-five graduate/undergraduate, French-speaking students at the University of Nantes (25 females, M age = 21.09 years, SD = 3.73, M education = 13.24 years, SD = 4.54). We evaluated verbal episodic memory of the participants with the test of Grober and Buschke [20], with the maximum score being 16 points. The mean score of the participants was 12.16 (SD = 2.21). Additionally, working memory was evaluated through span tasks, where participants repeated strings of numbers in both forward and backward order. The mean score of the participants was 7.31 (SD = 1.92) on the forward span and 5.31 (SD = 1.28) on the backward span. Based on these memory assessments, we excluded four participants who performed two standard deviations below the norms on the Grober and Buschke [20] test, as well as one participant who scored three points below the norm on the backward span task. Furthermore, four participants with a history of psychiatric/neurological disorders and seven participants experiencing difficulties with eye movement recording were excluded. After applying these criteria, the final sample comprised 45 participants. This sample size was predetermined using G*Power [21] [within-subject measurement *t*-tests as our experimental design involved two repeated conditions (i.e., near vs. distant future), 95% power, an estimated probability of making a Type I error as 0.05, and a medium effect size of 0.25 [22]]. In the final sample, no significant differences were observed regarding gender [*X*^2^ (1, *N* = 45) = 0.55, *p* = 0.45]. The study was validated by the ethic committee of the University of Nantes (reference IORG0011023).

### 2.2. Procedures

**Figure 1:**
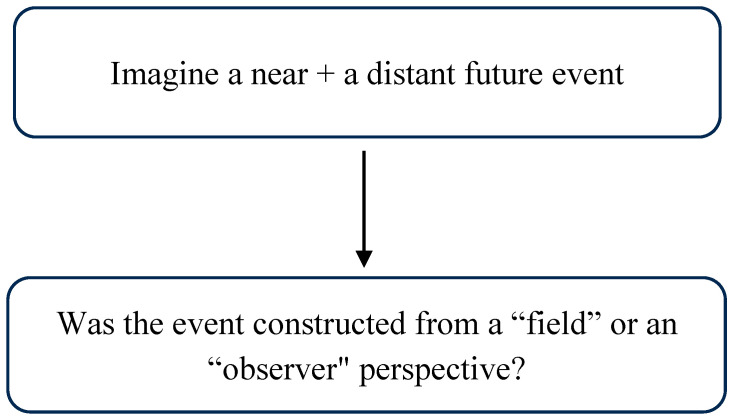
Illustration of procedures.

The procedures are illustrated in Figure 1.

#### 2.2.1. Future Thinking and Eye Movement Recording

Participants were instructed to verbally envision two future events: one occurring in the near future and the other in the distant future. While narrating these events, participants wore eye-tracking glasses and faced a blank white wall. They were prompted to be specific about the details of each event, including where it would take place, who would be involved, and their emotional experiences. Following the protocol outlined by D’Argembeau and Van der Linden [10], participants were given two minutes to describe each event, with the duration clearly communicated to aid in structuring their narratives. The order of the near and distant future trials was counterbalanced across participants. Participants wore eye-tracking glasses (Pupil Lab) with a gaze position accuracy of <0.1° and a 200 Hz sampling rate. These glasses emit near-infrared light onto the eyes, detecting and tracking pupil and corneal reflection and, thus, providing precise measurements of gaze direction and fixation points. Eye movement data were recorded and processed using the Pupil Capture software V 3.5.1. Calibration of the eye-tracking glasses was conducted before each trial by instructing participants to focus on a black cross positioned at the center of the wall. The experiment took place in a quiet room at the psychology department of the University of Nantes, with consistent lighting conditions and participants seated approximately 30 to 50 cm away from the wall. They were instructed not to look beyond the wall but were free to explore its entire surface. Unlike previous research utilizing screen-based designs, our study employed a wall-based setup to allow for unrestricted visual exploration, minimizing constraints on the visual system.

#### 2.2.2. Mental Visual Perspective

We replicated the procedures of previous research using the “field/observer” paradigm [23,24], as originally described by Nigro and Neisser [9]. Following the near and distant future thinking tasks, participants were briefed on the concept of visual perspective. Specifically, they were informed that when imagining an event, they might perceive it from either a field or an observer perspective. We clarified that the field perspective entails visualizing the event from a first-person viewpoint, as if experiencing it through their own eyes. Alternatively, the observer perspective involves visualizing the event from an external viewpoint, where they may see themselves within the scene, along with their surroundings. Participants were then prompted to verbally indicate their chosen “field/observer” perspective for each imagined future event.

#### 2.2.3. Dependent Variables

In alignment with prior studies [14,25], we analyzed several eye movement parameters, including fixation count, fixation duration, saccade count, saccade duration, and total saccade amplitude. Fixation and saccade counts were normalized to per-minute rates due to variations in the duration of future thinking tasks among participants. Fixation and saccade durations represented the mean duration of fixations and saccades, respectively, measured in milliseconds. Total saccade amplitude denoted the overall angle covered by saccades during the task. Additionally, blinks were automatically excluded from the dataset, and gazes exceeding a horizontal deviation of 2° were excluded (comprising 4.8% of the dataset). To assess reconstruction time consistently between conditions, we measured the total duration of recording. Furthermore, we tallied the frequency of “field” and “observer” responses provided by participants for both near and distant future thinking tasks.

#### 2.2.4. Statistical Analysis

Regarding eye movement (scores are provided in Table 1), we compared each dependent variable (i.e., number of fixations, fixation duration, number of saccades, saccade duration, total amplitude, and duration of recording) between near and distant future thinking using the paired samples *t*-test. Regarding mental visual perspective, we compared the number of “field” and “observer” responses (provided in Table 2) between near and distant future thinking with chi-square tests. We provided effect sizes by using Cohen’s d [22]: 0.20 = small, 0.50 = medium, 0.80 = large. Level of significance was set as *p* ≤ 0.05.

### 2.3. Results

#### 2.3.1. Fewer, but Longer, Fixations for Near Than for Distant Future

Compared with distant future thinking, near future thinking triggered fewer fixations [*t*(44) = 3.94, *p* < 0.001, Cohen’s *d* = 0.73] but longer fixation duration [*t*(44) = 2.77, *p* = 0.008, Cohen’s *d* = 0.56]. No significant differences were observed between near and distant future thinking regarding number of saccades [*t*(44) = 0.87, *p* = 0.39, Cohen’s *d* = 0.16], duration of saccades [*t*(44) = 0.44, *p* = 0.66, Cohen’s *d* = 0.08], or amplitude of saccades [*t*(44) = 1.15, *p* = 0.25, Cohen’s *d* = 0.22]. No significant differences were observed between near and distant future thinking regarding duration of recording [*t*(44) = 0.36, *p* = 0.72, Cohen’s *d* = 0.08].

#### 2.3.2. More “Field” Perspective Responses for Near Than for Distant Future

Compared with distant future thinking, near future thinking triggered more “field” [*X*^2^ (1, *N* = 47) = 6.15, *p* = 0.013, Cohen’s *d* = 0.78] and fewer “observer” responses [*X*^2^ (1, *N* = 47) = 6.49, *p* = 0.011, Cohen’s *d* = 0.80]. Near future thinking triggered more “field” than “observer” responses [*X*^2^ (1, *N* = 45) = 8.02, *p* = 0.004, Cohen’s *d* = 0.93], whereas distant future thinking triggered fewer “field” than “observer” responses [*X*^2^ (1, *N* = 45) = 6.15, *p* = 0.01, Cohen’s *d* = 0.79].

## 3. Discussion

We investigated eye movement and mental visual perspective during near and distant future thinking. Analysis demonstrated fewer but longer fixations for near than for distant future thinking. Analysis also demonstrated more “field” mental visual perspective responses for near than for distant future thinking. These findings demonstrate, for the first time, how the temporality of future thinking triggers distinct eye movement activities.

To begin with the eye movement activities as observed in our study, near future thinking triggered fewer fixations and longer fixation duration during near than during distant future thinking. In other words, when constructing near future events, participants demonstrated fewer, but longer, fixations than when constructing distant future events. This pattern of fixations can be attributed to the complexity of mental visual processing during near and distant future thinking. Generally speaking, the duration of fixations reflects the allocation of attention to a given stimuli [26] and stimulus complexity [27]. Furthermore, the duration of fixations reflects information processing [28]. Therefore, the long fixations during near future thinking may mirror a mental visual exploration involving processing of a more complex visual representation compared with distant future thinking.

We suggest that the long fixations during near future thinking can mirror the processing of complex visual representation. Our assumption can be supported by the Construal-Level Theory [29,30,31]. According to this theory, the construction of near events (e.g., travel next month) typically triggers a concrete and context-rich representation (e.g., booking a convenient hotel) compared to distant events, which typically imply a decontextualized representation that conveys only the gist or general meaning of the event (e.g., enjoying next year’s holidays). In other words, as future events become temporally distant, representations become more abstract, probably because people typically possess limited information about the distant future events. Supporting this model, research has demonstrated that, compared with the near future, distant future self-representations are typically more abstract [32]. For example, distant future self-representations typically involve less complexity and a greater degree of schematicity compared with near future self-representations [33,34]. By demonstrating how near future thinking involves a concrete and context-rich representation, the Construal-Level Theory supports our attempt to associate the long fixations during the construction of near future events to the complex visual representation of these events.

The discovery of fewer but longer fixations during near future thinking holds significant implications for understanding cognitive processes in future thinking. Our findings suggest that temporally proximal events prompt individuals to engage in more elaborate mental simulations, involving detailed contextual information and sensory-rich representations. Moreover, the identification of distinct eye movement patterns corresponding to different temporal distances provides insights into the temporal dynamics of mental imagery and perspective-taking processes. These findings may have practical applications in various domains, including education, marketing, and clinical psychology, by informing interventions aimed at promoting forward planning, decision-making, and goal-setting behaviors. Moreover, the consequences of our findings extend beyond theoretical insights into future thinking. By elucidating the relationship between temporal distance and eye movement patterns, our study opens avenues for practical applications in various domains. For instance, in education, understanding how individuals mentally simulate near and distant future events can inform instructional strategies aimed at enhancing long-term planning and goal-setting skills. In marketing, insights into the cognitive processes underlying future thinking can guide the development of persuasive messaging and advertising campaigns that resonate with consumers’ temporal perspectives. Additionally, in clinical psychology, knowledge of how temporal distance influences mental imagery can inform therapeutic interventions targeting future-oriented cognition in individuals with mood disorders or anxiety. Overall, our findings not only advance our understanding of future thinking but also offer practical implications for promoting adaptive decision-making and behavior across diverse contexts.

Besides being associated with the complexity of visual representation, the long fixations during near future thinking can be associated with the first-person perspective. As demonstrated in our study, most near future events triggered a “field” mental visual perspective, replicating previous research on the prevalence of this perspective during near future thinking [10,11,12]. The “field” perspective can be associated with the vividness of the visual representation of near future events. Generally speaking, this perspective involves imagining the event as it being experienced in the reality [9]. Furthermore, this perspective, as typically observed during near future thinking, involves the construction of vivid mental images [35]. Therefore, and because the “field” perspective has been associated with the vividness of the retrieved mental image, it is tempting to consider the long fixations, as observed during near future thinking, as a behavioral indicator of both the “field” perspective and its vividness.

Although it is tempting to propose that the first-person perspective, as observed during near future thinking, can be expressed in terms of long fixations, this suggestion should be considered with some caution. While our study demonstrates that near future thinking triggers both long fixations and “field” responses, we provide no evidence that the eye movement activities during future thinking (i.e., the few and long fixations during near future thinking, or even the non-significant differences between near and distant future thinking in terms of saccades) are the result or origin of mental visual processing. As mentioned in the introduction here (see above), eye movement in general has been considered an index of visual imagery during memory retrieval [15,16,36,37]. Research has also attributed eye movement during future thinking to the attempt by the visual system to find (through saccades) and activate (through fixations) stored mental representations [14]. While prior research, as well as our current study, provides a new behavioral insight, in its current state of development, this research only demonstrates how past and future thinking trigger both specific mental visual perspective and specific eye movement patterns (e.g., near future thinking triggers both “field” responses and long fixations, as demonstrated in our study). That being said, this research provides an exciting and novel departure from the traditional research widely assessing mental visual imagery during both past and future thinking in terms of subjective experience, by adding methods to measure eye movements.

Traditional research on mental visual imagery during both past and future thinking has demonstrated not only how the mental visual perspective (i.e., first- vs. third- person) can be influenced by temporality (i.e., near vs. distant events) but also how this perspective can be influenced by emotion. For instance, trauma memories have been widely associated with a third-person perspective [38], probably because this perspective involves avoidance and is less affect-provoking [39]. Further, the first-person perspective has been considered a predictor of the severity of post-traumatic stress disorder symptoms up to one year after the traumatic event, probably because this avoidance strategy plays a role in the maintenance of trauma [40]. The same thing can be said for depression, as the retrieval of negative memories from a third-person perspective has been linked to maladaptive avoidant strategies [41,42]. Therefore, it would be of interest to evaluate whether the third-person perspective during memory retrieval in post-traumatic stress disorder and depression can be associated with a specific pattern of eye movement activities. It would be also of interest to investigate this issue for future thinking because post-traumatic stress disorder, depression, and, critically, anxiety, have been associated with taking a third-person perspective during future thinking [43]. In our view, the assessment of eye movement during past and future thinking in these clinical disorders will provide a valuable behavioral indicator of cognitive processing in patient populations.

One limitation of our study is the assessment of only one near and one distant future event. Future research can replicate our study using a large number of near and distant future scenarios. As suggested previously, future research should also replicate our study on clinical disorders (e.g., post-traumatic stress disorder, depression, and anxiety) as these disorders have been associated with deficits in visual perspective. Furthermore, while our study utilized the field/observer paradigm to assess visual imagery, it would have been intriguing to incorporate direct manipulation of visual imagery. For example, inviting participants to construct concrete versus abstract conceptions of the future could have provided valuable insights into eye movement during future thinking.

It would be valuable to situate our findings within the context of recent advancements in computer vision research. For instance, Yang, et al. [44] conducted a thorough review of human parsing, which involves partitioning humans in images or videos into semantic parts. Their study sheds light on the challenges and potential research directions in this field, providing insights into how individuals mentally parse and integrate visual information during cognitive tasks such as future thinking. Additionally, Singh, et al. [45] introduced Latent Graph Attention, a framework for efficiently incorporating a global context into image processing tasks. This advancement aligns with our investigation into how individuals construct mental representations of future events, as it facilitates the integration of contextual information across spatially distant points in images. By aligning our findings with these developments in computer vision, future research stands to benefit from a multidisciplinary perspective that enhances our understanding of the cognitive processes underlying future thinking and mental imagery. Moreover, future replications could leverage recent advancements in machine vision optics. Innovations such as light-trapping-structure vertical Ge photodetectors enable high-speed and high-sensitivity detection of optical signals, thereby improving the precision of eye movement analysis during future thinking [46]. Additionally, advancements in optical computing architectures and adaptive multiscale imaging mechanisms offer opportunities for real-time analysis of eye movement data with enhanced accuracy and efficiency [47]. Moreover, the integration of on-chip metasurfaces in augmented reality holography presents new avenues for immersive and multifunctional augmented reality displays [48], potentially enhancing gaze-based interactions and personalized content delivery. Incorporating insights from these advancements into eye-tracking research could lead to significant improvements in understanding eye movement patterns during future thinking tasks.

To summarize, research on the mental visual perspective during future thinking has mainly investigated this perspective in terms of subjective measures. Our current study provides an exciting and novel departure from this research by proving new behavioral insight into the mental visual perspective during future thinking. While research on eye movement and past and future thinking in general stills in its infancy in terms of available empirical studies or even established models, we believe that this generation of research will yield new insights into the relationship between eye movement and cognitive characteristics of both past and future thinking.

## Figures and Tables

**Table 1:** Characteristics of eye movements during near and distant future thinking.

	Near Future	Distant Future
Fixation count per min	57.38 (17.23) *** (less fixations in near than in distant future thinking)	79.11 (29.23)
Fixation duration in ms	794.36 (510.69) ** (longer fixations in near than in distant future thinking)	564.41 (216.64)
Saccade count per min	62.69 (28.10) ^ns^	66.82 (23.26)
Saccade duration in ms	55.70 (27.34) ^ns^	53.36 (19.16)
Total amplitude of saccades	1622.02 (857.69) ^ns^	1425.45 (630.14)
Duration of recording in msec	82,604.34 (21,032.63) ^ns^	83,311.29 (25,162.89)

Note. Standard deviations are given between brackets; maximum duration of recording was 120,000 ms; differences were significant at ** *p* < 0.01, *** *p* < 0.001; ^ns^ differences were not significant.

**Table 2:** Number of “field” and “observer” responses during near and distant future thinking.

	Near Future	Distant Future
Field	32 *	15
Observer	13 *	30

Note. For each condition (near and distant future thinking), the total number of “field” and “observer” responses was 45 as participants (*n* = 45) were required to provide either an “field” or an “observer” response; differences were significant at * *p* < 0.05.

## Data Availability

Raw data is available upon request to the correspondent author.
